# Understanding Public Attitudes Toward Researchers Using Social Media for Detecting and Monitoring Adverse Events Data: Multi Methods Study

**DOI:** 10.2196/jmir.7081

**Published:** 2019-08-29

**Authors:** Su Golder, Arabella Scantlebury, Helen Christmas

**Affiliations:** 1 Department of Health Sciences University of York York United Kingdom; 2 Leeds Teaching Hospitals NHS Trust Leeds United Kingdom

**Keywords:** adverse effects, social media, ethics, research, qualitative research, digital health, infodemiology, infoveillance, pharmacovigilance, surveillance

## Abstract

**Background:**

Adverse events are underreported in research studies, particularly randomized controlled trials and pharmacovigilance studies. A method that researchers could use to identify more complete safety profiles for medications is to use social media analytics. However, patient’s perspectives on the ethical issues associated with using patient reports of adverse drug events on social media are unclear.

**Objective:**

The objective of this study was to explore the ethics of using social media for detecting and monitoring adverse events for research purposes using a multi methods approach.

**Methods:**

A multi methods design comprising qualitative semistructured interviews (n=24), a focus group (n=3), and 3 Web-based discussions (n=20) with members of the public was adopted. Findings from a recent systematic review on the use of social media for monitoring adverse events provided a theoretical framework to interpret the study’s findings.

**Results:**

Views were ascertained regarding the potential benefits and harms of the research, privacy expectations, informed consent, and social media platform. Although the majority of participants were supportive of social media content being used for research on adverse events, a small number of participants strongly opposed the idea. The potential benefit of the research was cited as the most influential factor to whether participants would give their consent to their data being used for research. There were also some caveats to people’s support for the use of their social media data for research purposes: the type of social media platform and consideration of the vulnerability of the social media user. Informed consent was regarded as difficult to obtain and this divided the opinion on whether it should be sought.

**Conclusions:**

Social media users were generally positive about their social media data being used for research purposes; particularly for research on adverse events. However, approval was dependent on the potential benefit of the research and that individuals are protected from harm. Further study is required to establish when consent is required for an individual’s social media data to be used.

## Introduction

### Background

#### Adverse Event Mentions on Social Media

Patient reports of adverse drug events from social media have great potential to improve the detection and monitoring of adverse effects of medications. Increasingly, people are posting information on their experiences of adverse events on social media, including discussion boards and Twitter. An overall prevalence of adverse events reports on social media has been estimated at 0.2% on generic social media platforms such as Twitter to 8% of all posts in patient forums [1]. The order of magnitude of data and the speed at which the data are made available (approaching real time) make social media a tool with the potential to revolutionize drug surveillance. This has led to a massive surge in the development of techniques for social media analytics of adverse event posts [2] and the assessment of data on adverse events from social media [1].

#### Ethical Challenges of Social Media Research

However, these new research avenues are not without ethical challenges [3-7]. Potentially difficult considerations surround the purpose and value of the research, benefits and harms to participants as well as privacy, informed consent, and confidentiality [8]. The ethical issues of social media research have been much debated [3,8-11]. However, there has been little formal investigation of patients’ views on the use of social media for research purposes. We previously undertook a systematic review with 17 included studies, which aimed to ascertain attitudes toward the ethics of research using social media [8]. However, only 5 studies were specifically concerned with health-related research [8]. No studies explored the views of social media users on the use of social media for pharmacovigilance or identifying adverse events of nondrug interventions [8].

### Objectives

We aim to address this gap by exploring the attitudes of the public toward their social media posts being used to monitor adverse events for research purposes. More specific research questions include the following:

1. What are the views and experiences of social media users on reporting adverse events on social media?
2. What are social media users’ attitudes toward social media being used as a source for research data?
3. What are social media users’ attitudes and ethical concerns toward social media being used as a source for adverse event information?
4. What do social media users perceive to be the ethical barriers to the use of social media in research and pharmacovigilance?

## Methods

### Overall Approach

This qualitative exploratory study used interviews, virtual discussions, and a focus group to explore social media users’ views and attitudes toward the use of social media to monitor adverse events. A multi methods approach was selected to ensure that we obtained the perspectives from a varied sample, which was particularly important given the exploratory nature of the study and target population—members of the public [12].

### Interviews

Social media users are universally diverse and so to capture this diversity, we used a convenience sampling frame and 5 different methods to recruit participants to interviews.

#### Posters and Flyers

Posters and flyers advertising the study were displayed across the University of York campus and at community centers in York.

#### Local Facebook Groups

Recruitment advertising was undertaken on York-based Facebook groups.

#### University of York Staff Networks

Study information were posted in staff newsletters.

#### Local Networks

Local parents, breastfeeding networks, and exercise groups were contacted to identify potential participants. Previous research has shown these groups to have a higher response rate than patient forums [13].

#### Snowballing Technique

Participants were encouraged to invite friends and family to contact the researcher if they were interested in taking part.

Interviews were semistructured and followed a topic guide, which was based on previous literature identified in a systematic review and expert opinion [8]. To refine the topic guide, we conducted 3 pilot interviews with University of York staff who would have been eligible for the study. The topic guide included a *core* set of questions to give some consistency but was used flexibly to allow for new and unanticipated responses to be introduced (Multimedia Appendix 1: Interview topic guide). Interviews permitted conversations to flow as naturally as possible while ensuring that the following topics were covered: *social media and health information on the Web*, *reporting of side effects of drugs or other treatments*, *attitudes toward research and different types of research or researchers*, *privacy expectations*, and *research conduct*.

Interview participants were sampled until no new themes emerged, as we aimed for theoretical saturation [14]. A total of 24 face-to-face interviews were conducted by either SG or HC (Table 1). Each interview lasted between 15 and 45 min and was undertaken in York between April and September 2018.

**Table 1:** Participant characteristics.

Participant type	Participant total, N	Gender, n			Age (years), n			
		Male	Female	Unknown	18-30	31-45	46-65	≥65
Interviewees	24	5	19	0	13	3	5	3
Virtual discussants	20	13	4	3	NR^a^	NR	NR	NR
Focus group participants	3	0	3	0	0	1	2	0
Total	47	18	26	3	13+^b^	4+	7+	3+

^a^Not reported.

^b^Refers to the fact that the number might have been higher if the age of the virtual discussants had been available.

### Virtual Discussions

We contacted 7 patient forums (from general health to specific forums such as cancer or mental health forums) to obtain permission for virtual discussion. The individual forums are not listed here as we do not have permission to do so. Virtual discussions were stimulated by creating threads on selected social media sites and monitoring the Web-based posts responding to the threads (Multimedia Appendix 2: Posts to create Web-based discussion). Permission to begin discussion threads was granted by 3 of the 7 patient forum moderators, and these 3 threads resulted in 31 replies from 20 posters (Table 1). This allowed us to tap into different populations, as participants were not limited to the local geographical area, and provided an anonymous method of communication for individuals who might post on social media but were unwilling to participate in a focus group or interview. It was anticipated that those posting on health information sites might be more likely to have had posts used in research and, therefore, might hold a different perspective to those recruited through interviews and focus groups. There were no limits on the number of participants and discussions were open for 3 weeks in August 2018.

### Focus Group

A focus group was conducted to encourage spontaneous generation of ideas through group dialog and interchange via a series of 8 statements and 6 scenarios (Multimedia Appendix 3: Focus group topic guide). The 8 statements followed the same themes used in the interviews—*social media and health information on the Web*, *reporting of side effects of drugs or other treatments*, *attitudes toward research and different types of research or researchers*, *privacy expectations*, and *research conduct*. The scenarios, on the other hand, suggested research on side effects being undertaken by different types of researchers or institutions, with different intentions, types of data collected (eg, numerical vs textual quotes), and social media platforms.

The focus group took place in December 2018 and lasted 60 min. A total of 3 mothers, recruited from a baby group at a Sure Start Community Centre, who met approximately 6 times a year took part in the focus group (Table 1).

### Approval Process

Ethical approval was obtained from Department of Health Sciences’ Research Governance Committee at the University of York. Written informed consent was obtained from all participants at the start of the focus group and interviews. At the beginning of each interview and focus group, verbal consent was obtained, assurances regarding participants’ anonymity and confidentiality were made, and participants were informed that they could withdraw from the study at any time. All participants were provided with a participant information sheet before providing consent.

### Analysis

The interviews and focus group were audio recorded and transcribed verbatim. All participants were assigned pseudonyms for data reporting and analysis. Analysis was facilitated by use of the qualitative data management software package Nvivo (version 11, QSR International). Information from the interviews, virtual discussion, and focus groups is presented together throughout in the reporting of our results.

Initially, we analyzed the data from each data collection method separately, using the recommended stages of thematic analysis as described by Braun and Clarke, 2006 [15]: familiarization, generating initial codes, searching for themes, reviewing themes, defining and naming themes, and data reporting. Throughout the analysis, theme and subtheme development was largely deductive, based on the topic guide and in the second stage of the analysis, the underpinning framework. However, themes were also allowed to emerge.

We then undertook a second level of analysis, where our initial themes and subthemes from all data sources were mapped onto a framework that emerged from a recent systematic review of the ethics of social media research [8]. Adopting a 2-stage approach to our analysis enabled our findings to be translated from an initial list of descriptive themes to broader overarching ones and in doing so, placed the study findings in a broader context and in line with the current evidence. For transparency, Table 2 demonstrates how our initial themes map onto the framework. This framework was selected as it was the most comprehensive framework available, drawing on the results of 17 included studies, and adapted as necessary. The framework proposes 4 main influencing factors when considering whether individuals are willing for researchers to use their social media data to monitor adverse events: the research, the social media users, consent, and responsibilities (Table 2).

**Table 2:** Theoretical framework.

Themes and subthemes adapted from Golder 2017 [8]		Description
**Researchers**		
	Patients’ and/or members of the public’s views on researchers using social media	General reactions to the concept of using social media content for research on adverse effects
	What is the purpose of the research?	Overall outcome or intention of the research
	Who is conducting the research?	The affiliation of the researcher (such as university or commercial company)—this is associated with the perceived benefit of the research
	Quality of research using social media data	High or low quality of the methodology used, including risk of bias
	Potential harm to researchers	Any risks of harm that the researcher is exposed to
**Social media users**		
	Perceived risk of harm to social media users	Any risks of harm that the social media users are exposed to, either individually or as a group
	Perceived risk of harm to vulnerable groups	Any risks of harm for particular groups—groups could be determined as vulnerable by either their individual characteristics or the topic discussed
	The original intended purpose of the posts	The intent of the poster at the time the message was placed
	Privacy on social media	The public versus private nature of social media and the need for anonymity or confidentiality
**Consent**		
	Informed consent	Permission for posts to be used in a study—this includes issues around the terms of service (also known as *terms of use* or *terms and conditions*). These are the rules agreed to for using social media sites
	Research disclosure	Researchers being transparent and honest about their intent—this can be either up-front or at a later stage
**Responsibilities**		
	Social media user	The issue of self-regulation is whereby individuals control content through personal censorship
	Social media platform	The type of social media platform, for example, closed or open, personal or professional, or social norms connected with the platform
	Site administrators	Site administrators, list administrators, or list moderators are often in charge of maintaining a discussion or mailing list
	Governments	Refers to legal issues, regulation, or government oversight and includes issues of copyright

We adopted a reflexive approach to data collection and analysis. A total of 2 researchers undertook data collection (SG, HC) and analysis (SG, HC, and AS), with regular discussions held between the research team throughout data collection and code and theme development. The research team are considered to be in a neutral position in relation to any past expectations that may have influenced our ability to collect or analyze the data. SG is a researcher with a background in systematic reviews and social media research. HC is a Public Health Registrar. AS is a mixed-methods researcher with a background in qualitative evaluations in electronic health and systematic reviews.

## Results

### Presentation of Data

First, we present data relating to participants’ experience of using social media and reporting adverse side effects, which provide important context about the participants represented in our sample. The remainder of our study’s findings are then presented according to the 3 overarching themes outlined in the framework: the 2 actors (*researchers* and *social media users*), consent, and context (in terms of responsibilities, such as regulations or code of conduct), which provide insight into social media users’ attitudes toward social media data being used for research and the various challenges associated with this.

### Participants’ Experience of Using Social Media and Reporting Side Effects

#### Experience of Using Social Media

All participants used social media. However, younger interviewees (<30 years) tended to use more social media platforms and more frequently. For instance, younger participants discussed frequency of daily usage, whereas older participants discussed accessing social media on a times-per-week basis. The most commonly used social media platform was Facebook, followed by Twitter, Instagram, LinkedIn, and Snapchat. Other platforms included the following: Mumsnet, patient forums, Tumblr, Reddit, and YouTube. Participants reported using Facebook, largely for personal reasons, such as keeping in touch with friends and family, whereas platforms such as Twitter were largely for professional use.

Only 3 participants reported using social media to explore possible medication side effects. This was largely because the majority of participants had either not considered the possibility of doing so or had concerns about the trustworthiness of information on social media. Those who did use social media to access health information viewed it as a unique and valuable information source that provided access to information that was not accessible elsewhere. For example, information posted on social media was perceived to give a greater insight into people’s lived experiences than a *list of possible side effects*:

There’s a strong mental health community on Tumblr, and so I did search for a few tags about the withdrawal symptoms associated with this, because I was looking for more personal experiences rather than a list of possible side effects.

Rebecca, Female 18-30 Interviewee

#### Experience of Reporting Side Effects

None of the participants from the interviews or focus group had reported an adverse event to a regulatory agency (eg, the Yellow Card system in the United Kingdom). Although supportive of such official reporting channels, few had heard of them and many felt that they would have to experience a very serious adverse event to warrant using them.

Less than half of interview and focus group participants had mentioned a side effect on social media, with some of these individuals no longer willing to do so. Many considered side effects to be too personal or preferred to talk to a health professional, family member, or friend. This was largely because of confidentiality concerns, wanting an expert opinion, or viewing social media as unhelpful and in some cases scaremongering. Those that did discuss side effects on social media mostly did so to discuss the practical implications of experiencing side effects to converse with people with similar experiences or help others:

I’ve used sort of social media, mainly Facebook, to say, for example, I wanted to come off the medication and try another one, will that affect my driving?

Jamie, Male 31-45 Interviewee

I search the hashtag epilepsy a lot, if somebody’s put like “oh, I’m on Keppra...” which is a medication that I used to be on “...and it’s making me feel like this, does anybody else get this?” I will put “I was taken off that because of the same reason” or something like that.

Carol, Female 18-30 Interviewee

However, a proportion of those who were willing to discuss side effects on social media admitted that they would only discuss minor side effects that they did not feel warranted discussions with a doctor, or they were only willing to do so on a closed group:

Say you’ve got, something like breast cancer and you were wanting to deal with the side effects of that and you were part of a closed breast cancer support group. You could imagine in that case you might go, “oh my God. I’m finding chemo hard to deal with. What have other people done?” So I can imagine posting in a closed social media forum but not in an open forum.

Arabella, Female 31-46 Focus Group Participant

### Researchers

#### Patients’ and/or Members of the Public’s Views on Researchers Using Social Media

Although the majority of participants were positive about the idea of their social media data being used for research purposes, 1 interviewee and 3 virtual discussants were *completely against it* and felt social media data should never be used for research in any circumstances. Even of those that were supportive of the use of their social media data for research purposes, a sizable proportion felt that their approval would depend on certain factors, such as the purpose and quality of the research and who is conducting it.

#### 

The purpose of the research was considered one of the main factors influencing whether participants would approve the use of their social media data for research purposes. Participants were particularly supportive of their data being used for research where it was clear that there would be a societal benefit and liked the idea of helping others. Health care research, and in particular pharmacovigilance, was seen as a *good thing* over and above other types of research, with potential benefit to others. Participants felt that the ubiquitous use of social media, and consequently, large amount of unique, valuable, and accessible data, meant that it could be used to improve *patient safety*, *save lives*, and help *pick up side effects quicker*. For example, participants discussed how social media made side effect information more readily available, and it was felt that some people might be more willing to post about their side effects on social media than report them to a health professional or through traditional channels:

It could be very beneficial because you could be finding out side effects that aren’t so readily reported to health professionals.

Imogen, Female 18-30 Interviewee

I guess it’s useful in a way that people would post on social media to report what’s happening in their daily lives...maybe it’s easier to post on social media rather than talk to the doctor, cos they don’t have to make a trip to the doctor’s.

Carla, Female 20 Interviewee

This extended to data on medication adherence and rare adverse events for which social media might be able to uncover information more easily:

There must be loads of people that get side effects and rather than going and tell anyone about it, they just stop taking the medication. So, nobody would ever know. And they are more likely to respond and say, “oh yeah—that happened to me and then I stopped taking it.” So, there could be millions of people that are getting the same side effect, give up on the tablet and then just don’t say anything. So, I think, it probably could be a really good way to find those people that are hidden that haven’t reported it officially.

Kate, Female 46-65 Interviewee

#### Who is Conducting the Research?

Participants were divided as to whether they thought that the affiliation or type of researcher (such as an academic, a government official, or an industry representative) made a difference to the ethical implications of research using social media data. Although some felt it did not matter, as researchers were *all working toward similar goals* and thus would *put them in the same pot*, others categorized researchers according to the underlying motives of their research. The biggest issue was whether the motives were for profit, and participants often stated that they would be less supportive of commercial organizations because of their *vested interests*, whereas *altruistic* motives were met with greatest approval:

The only one I would not like would be commercial. If you’re trying to make life better for people, then by all means, if you’re trying to generate profit, then no I think that’s not acceptable.

Jamie, Male 31-45 Interviewee

Participants also raised concerns about the motives of research undertaken by the following: Government, charities, and students, who were described as having their *own agenda*, influencing public opinion, or obtaining a degree or qualification. Participants tended to be more comfortable with academic institutions, which were viewed as more likely to adhere to good ethical conduct and not have ulterior motives:

Anybody who is making any kind of income I’d be very unhappy about. So, strangely enough, I would actually feel more comfortable if it was an academic researcher, because I would hope that people had some awareness of research ethics. And weren’t particularly getting any direct benefits from it, because I assume for a lot of academic researchers whether 100 people said, this was rubbish, and, 100 people said, “this was really good”, it doesn’t actually matter to them. So, they haven’t got a particular axe to grind.

Megan, Female 46-65 Interviewee

I would have much more faith in an academic institution or institutions than I would in a corporate company. I think I would trust academic institutions more than I would the government to be setting up anything. I just feel like there is much more pressure on the government to behave in ways that, God, I sound like my parents! I do think there is that pressure to benefit the corporations and the big conglomerates more than potentially there is to protect the individual privacy or just kind of adhere to basic ethics.

Gill, Female 31-45 Interviewee

#### Quality of Research Using Social Media Data

The quality of social media content was a particular concern. Some participants used the internet for health information but preferred trustworthy sites, such as National Health Service (NHS)–managed sites rather than social media sites. The validity of any research conducted using social media data was bought into question, and this was considered to be a particular concern when the research related to important issues, such as health or side effects. It was acknowledged that social media might not reflect the truth as it was considered easier to make false claims or exaggerate the truth on social media than to a health professional, and people’s motives for posting might reflect the content. For example, people may post to try to provoke a reaction or particular response. In addition, participants were wary of side effects being wrongly attributed by the public to a particular drug and cited the measles, mumps, and rubella vaccine and autism as an example:

I don’t trust the common layperson...I think people are much too willing to see a causal link where there isn’t one and that especially with something like this. This would really worry me.

Gill, Female 31-45 Interviewee

I find that quite worrying that they base research on what people post because I don’t always think what people post is necessarily the truth and may exaggerate their possible drug use or it might be that they exaggerate their symptoms of an illness. So, I don’t think a forum like Facebook or something like that would be very reliable.

Mandy, Female 31-45 Interviewee

There is loads of issues about going just to social media—are all these people telling the truth? How representative are they? Why should I believe them? Are they real? Do these people really exist? Is what they’re saying true?

Megan, Female 46-65 Interviewee

Another concern with the validity of social media research related to the representativeness of social media users to the general population. It was thought that a lot of people did not use social media or, if they used it, did not post on health issues, which might mean that data were *skewed*. Participants felt that individuals who posted on social media might be more likely to have had either extremely positive or negative experiences or represented the more extreme people in society. Social media users were also perceived to be younger than the general population, and this was seen as a particular problem when studying health conditions that were more prevalent in elderly populations. People with some types of conditions were also thought to be more active social media users and more vocal than people with other conditions:

If I was reading a study that was like “we took information from social media”, I’d be cautious of reading it because with social media you’re only getting a percentage of the population. Are you getting like a broad enough snapshot of the population to get a proper sort of outcome? It’s not like going door to door, right, where everyone has a chance to have their say.

Helen, Female 18-30 Interviewee

A potential solution to the low validity of the research data when using social media was to use these data as an adjunct to other sources of information to provide a *rough idea* for background information:

I suppose it gives them a rough idea. But, it’s the fact that people exaggerate. It’s not controlled, is it? You know, I guess, it’s good for them to use maybe for background information to start work. But I wouldn’t see it as a reliable source to produce some findings from. Because of that fact that people exaggerate...So, I guess, I feel like it’s a really good way to get an idea of trends in things. But, shouldn’t be the only sort of thing for research. There should be other sources to back it up.

Kate, Female 46-65 Interviewee

### Social Media Users

Themes discussed by participants relating to *social media users* were concerned with the potential risks or harm to social media users and, in particular, vulnerable groups.

#### Perceived Risk of Harm to Social Media Users

Although some participants emphasized how the public nature of social media meant that there was little or no harm in the posts being used in research, others felt that the risks to social media users should be considered even if the data were in the public domain. Any risks of harm to social media users were perceived to be dependent on the type of information posted, with personal or sensitive information, such as side effects, warranting the need for more stringent ethical safeguards than opinions on trivial issues, such as television programs:

Even if it’s completely open, these people who are doing the research still need to think through what the implications are and think very carefully about how they’re using that information even when there’s no deceit required to get that information. I still think they need to think about the possible implications.

Arabella, Female 30-45 Focus Group Participant

#### Perceived Risk of Harm to Vulnerable Groups

Particular groups of people were perceived to be at greater risk of harm from research using social media content. Vulnerability was recognized in terms of age. The younger participants tended to perceive the older generation as more vulnerable because of a lack of understanding of how social media worked, as they had not grown up with it. On the other hand, the older generation perceived the younger people as more likely to share personal details without considering the implications of what they are posting and not listen to warnings from older generations. It was also acknowledged that irrespective of age, people could be *naïve,* and there was a danger of *getting carried away in a conversation* and forgetting how public social media posts were:

I’ve got a lot of friends who have been in the Retreat hospital, like the mental hospital, and I follow all of them on Instagram and Facebook and they very often have discussions how they’re feeling on the medication and so on. And then when they’re discussing it with each other sometimes I’m looking at it and I’m thinking, I don’t think everybody should be knowing that. That’s quite personal. And I think once they start a conversation they forget that the world can watch and they continue with it and you get caught up in it and you forget that actually everybody can read that now.

Kate, Female 46-65 Interviewee

Vulnerability was also linked to people’s illnesses or conditions, with some conditions or topics, such as side effects or mental health, considered more sensitive than others:

If someone has disclosed something about a mental health issue or something which is more potentially stigmatized, particularly something like HIV or something like that, I think you would have to be more careful about using that data because I think, they’re going to put themselves, potentially, in a more vulnerable position than if someone has had a bout of chicken pox or the flu.

Claire, Female 31-45 Interviewee

Users of patient forums were viewed as vulnerable by interviewees and focus group participants. This was reinforced by the virtual discussants, who considered themselves to be vulnerable and felt that they had enough to contend with already, stating “it’s hard enough to manage what we have—I don’t think we require any voyeur over that period” (Male, virtual discussant, paraphrased). Indeed, knowing researchers were looking through their posts may be very harmful to some virtual discussants who even stated that this would have put them off posting:

This discussion forum was my only method of connecting with individuals who were experiencing the same illness as me...Given I was debilitating paranoid, in the event that I had known researchers were potentially trawling posts for information, I doubt very much I would have posted.

Unknown gender, virtual discussant, paraphrased

#### The Original Intended Purpose of the Posts

The original purpose of the posts was mentioned by a few participants who were keen to emphasize that their reasons for using social media included the following: for support, to encourage open discussions on important issues, to help benefit others, or to find or communicate with followers, friends, and family. Ethical issues were, therefore, seen to be associated with their social media data being used for research purposes, particularly when considering that this was not one of the motives for posting and the fact that many people might not even be aware that researchers could use the data. Associated with this were concerns that their data might be taken out of context or misinterpreted:

If you put a public profile up...you weren’t thinking about researchers using your statuses, you were thinking oh maybe I’ll find more friends that way. Like maybe people will be able to find me better. So, I feel like it’s not valid to say that because it’s public there are no ethical issues because people weren’t intending for you to use their data in that way.

Sophie, Female 18-30 Interviewee

Maybe it is public but then they’re putting it in a public domain for a certain reason and then as a researcher you’re changing that use. So, that’s like me going into an interview and telling people that this is for research and then going off and actually using it for my own private company, you know, that’s not ethical so, it’s sort of a similar thing. I know it’s not the same but it’s got similar, kind of, parallels, in a way because you’re changing what that person did with that information...a researcher comes along and they take that information and use it in a completely different light to what it was originally intended. So, I think that, for me, is the main ethical issue with it. That intent and reformulation of that information. But, not to say, it shouldn’t be done.

Imogen, Female 18-30 Interviewee

#### Privacy on Social Media

Many participants were fully aware that they did not know how much of the information they posted was public and who could see or use their data. Participants were also aware that privacy settings existed but did not always keep these up to date or know what they were currently set as. Many also stated that they would like to know more about how privacy worked on social media but understood that this was a complicated issue:

I think it’s quite complicated to understand. You really have to make an effort to know what’s private and what’s not, because it’s just not as easy as that, because on Facebook you can send private messages to people, which are obviously only for that person, or you can send it in a big group, or can you make parts of your profile or the messages you post private and other parts not. So, I think you have to really make an effort yourself to know what you’re sharing with people and what you’re not sharing, and who with, and what parts of social media are accessible to different people.

Joanne, Female 18-30 Interviewee

I’ve only recently sort of found the privacy settings. So, yeah—I’ve had a Facebook page for a few years and it was only last year that I realized that it was going public. And then I changed the settings.

Mandy, Female 31-45 Interviewee

The difference between an in-person conversation and a conversation on social media was also highlighted, as face-to-face conversations even in public spaces were seen as more private than social media. Furthermore, although some participants considered all social media data to be public in one way or another, others considered there to be different ethical considerations depending on the social media platform. The more public the platform, for example, the more likely the participants were to agree that research was ethical on these sites. Concern was also raised that researchers might be able to access private accounts or areas of social media that individuals considered to be private:

All of them are very public so it doesn’t matter and on Facebook you’ve fifty or a thousand friends, it’s still public. I mean, I know people who say whatever you post on Facebook it’s yours, it’s private, but it really isn’t.

Evie, Female 18-30 Interviewee

### Consent

The issue of *consent* was discussed with emphasis on when and how informed consent was required and whether research disclosure was necessary.

#### Informed Consent

Whether informed consent is necessary for researchers to use an individual’s social media data was met with uncertainty and polarized opinions. A small number of participants felt that informed consent was a requirement for any research and that using *any information or observations from individuals without their knowledge or permission is unethical.* For the majority of participants, whether informed consent is required depended on a number of issues. The most frequently discussed factor was whether anonymity could be retained. This was seen as crucial to protecting the individual poster. Linked to anonymity and identity was the use of quotes. Although participants were accepting of quantifiable data (such as 63 people reported suffering from insomnia after taking drug X), they were less accepting of researchers using direct quotes that could lead to people being traceable, for example, by a simple Google search. It was, therefore, recommended that informed consent be obtained for direct quotes or that quotes are paraphrased.

Another important issue impacting on whether informed consent was considered necessary was whether the post was public or not and the type of platform it was posted on. It was generally accepted that informed consent was less of an issue for a social media platform such as Twitter than Facebook. Others also talked about the sensitivity of the data and how this might impact on whether informed consent was deemed necessary. For instance, it was not thought necessary to obtain consent for trivial information but data discussing health issues were considered more likely to require informed consent:

That’s a really interesting question, because you’re putting that information out there for the whole world to see...Depends what kind of...oh, I don't know, what kind of research they were doing maybe?

Carol, Female 18-30 Interviewee

The logistical problems of obtaining informed consent were acknowledged and described as an *utter nightmare*. This was both in terms of the number of people who would need to be contacted and beliefs that direct messaging was intrusive and unlikely to get a response.

There was overwhelming agreement that terms and conditions of the social media site would not be an acceptable or effective way to seek informed consent. This was mainly attributed to the fact that no one ever reads them because of the size of the print and their length, people are already *bombarded* with too much information, and they are constantly updated and revised. It was also pointed out that it would be very difficult for consent to be covered by the terms and conditions because of the variety of research that could be conducted. For instance, a social media user may be happy for their data to be used in some research but not in other research, and it would be impossible for all scenarios to be covered:

Conditions saying like your information can be used for research—what research? Who’s it going to, etc, there are so many different topics that you can research on social media that I might be fine with one but not the other, so I think to that extent, it’s a totally an unfair condition clause in my eyes.

Helen, Female 18-30 Interviewee

Participants proposed an *opt in* or *opt out* option as a potential alternative to relying on terms and conditions. For some individuals, concerns about the ethics and practicalities of obtaining consent led to suggestions for researchers to use social media to recruit participants or conduct a Web-based survey instead of using social media data:

I think there’s a number of reasons like a direct personal message wouldn’t work, cos like if someone’s going to reply to a message that was like I’m doing research on social media, I’d be like oh, this person’s bugging me, you know what I mean, I wouldn’t even reply to it. So, I think you might not get responses there, I think it’s just tricky with social media, but then again, how else are you going to ask for their consent.

Helen, Female 18-30 Interviewee

I suppose it depends on how many people you want to quote. For me, I’d be kind of wondering, potentially, can you contact the individual. But then, if you’re looking at 100/200 perhaps more people that is not going to be viable.

Gill, Female 31-45 Interviewee

#### Research Disclosure

The majority of participants felt that researchers should disclose their identity and the purpose of the research and believed that not to do so would be *sneaky*. However, there were occasions where disclosure was considered unnecessary, such as in situations where only numerical data are required, or where doing so would distort the research. It was thought that people might have changed what they said if they knew they were being studied, and thus different results might be obtained. It was also argued that other people on social media did not have to declare who they were. Indeed, part of the attraction of social media was perceived to be that it provided a space where people could pretend to be someone else or have a different persona to real life:

I guess nobody who is on there has to disclose who they are, do they? I mean they could be patients or they can be people who are just interested, or family, so I guess you don’t have to say that you’re a researcher...it’s nice to be more transparent, but then you might get different results. So yes, I think it’s not necessary, but it is a bit—a bit tricky.

Joanne, Female 18-30 Interviewee

Situations where research disclosure was seen as important included when it involved direct interaction with an individual as opposed to being observational:

If they’re just sitting and effectively using it to surf stuff that’s posted openly, then I’m not sure that if you had a Twitter account, I don’t think you need to say “social researcher @Twitter” kind of thing. But if you’re, if you’re effectively prompting people to get information. If you’re starting to pose questions saying, “has anybody got any experience of this?” At that point, to do that without being open about your background, that seems very deceitful.

Arabella, Female 31-46 Focus Group Participant

### Responsibilities

The issue of whose *responsibility* it is to oversee research practice and protect users was also discussed by the participants. Responsibilities tended to fall directly to the social media users, site (including the platform, such as Twitter or Facebook, and site administrators), or regulations.

#### Social Media User

Self-regulation was mentioned by many participants as the answer to protecting individual privacy and thus moving responsibility to the social media users. Some participants felt strongly that it was the responsibility of social media users to self-regulate, and this was largely attributed to the *voluntary* nature of posting:

If people are stupid enough to put really personal things out there and people see it then it’s your own fault. It’s fair game.

Harry, Male 18-30 Interviewee

If it’s out there, it’s out there...If you put it on social media, it’s there for everybody. That’s it.

Hilda, Female over 65 Interviewee

Some participants felt that posting about health issues was a strange or *somewhat bizarre* thing to do and could not understand why others might do so:

I’m astonished that people do [post on their health]. I find that incredible that something so personal people are quite happy to post questions online about it.

Megan, Female 46-65 Interviewee

Many participants also stated that over time they had changed their Web-based behavior. Although in the past they might have been more open and trusting of social media, over time they had become more cautious resulting in increased self-regulation:

I have in the past when I was younger, I think, I posted more but that was, I think, before I learned how often things go wrong.

Gill, Female 31-45 Interviewee

Participants had also become more cautious over time after hearing negative stories about social media from friends and family and/or in the media and made particular reference to media coverage of Cambridge Analytica:

In the past I’ve used it for work as well but as sort of, you know, you hear news stories about somebody has said something on social media and it’s got them into trouble at work, even though it’s nothing to do with work so, as I’ve got older and become a little bit more wary of that sort of thing.

Jamie, Male 31-45 Interviewee

Some participants also stated that they were likely to self-regulate more after taking part in this research, considering the issues around researchers using social media data:

I am slightly concerned now how open my Facebook page might be and how it’s being used—it would definitely make me be cautious of what I post.

Mandy, Female 31-45 Interviewee

#### Social Media Platform

Although terms and conditions of the social media platforms were rejected as a means to protect individuals, careful selection of social media platforms was considered important. The majority of participants differentiated between different platforms in terms of the ethical considerations of researchers using social media. Participants spoke about the unwritten rules or purpose of different types of social media. For example, Twitter was thought of as a broadcasting platform to publicize information and described as a *way of deliberately grabbing attention* or *posting out to the world* and was, therefore, perceived to be open for use by researchers. In contrast, Facebook was thought of as a place to share information with family and friends, with even public information on Facebook not necessarily considered open for use by researchers:

I feel like it’s different on different social medias because Twitter is less personal and more kind of flaunty, you post to everybody on Twitter...I think it’s different on Twitter than it is on Facebook because people are not talking to their family and their friends on that, they’re just writing things that they want others to see. So, I don’t see such an ethical problem of using people’s data from Twitter, as I do people using their stuff from Facebook.

Sophie, Female 18-30 Interviewee

I think something like Twitter you’re posting out to the world...if you’re posting on a public forum, for example, the BBC News Have Your Say, your comment is going on a forum that anyone in the world can see. If you’re doing it on Facebook, where you’ve explicitly said I want this group, these people to see my data, see my opinion or see my, you know, about side effects, then that’s strictly controlled, then I think that, yes, that’s the difference to me...Facebook’s different but Twitter, yes, Twitter’s the one where you’re posting out to the world.

Jamie, Male 31-45 Interviewee

#### Site Administrators

Site administrators or moderators were rarely mentioned. However, a couple of participants felt that researchers should disclose who they were to the administrators or moderators and ask permission from them to use the site in research.

#### Governments

Laws on social media research were generally seen as a positive by moving responsibility to governments. However, there was understandable confusion about what laws or regulations already existed and whether they could be applied to research using social media data. Ownership and copyright were also mentioned, and it was recognized that once posted, the social media platform might have ownership rights over information, with the user unable to reclaim what they posted. Although this was considered a cause for concern, participants did not see a way around this.

Participants were unsure whether there should be strict rules and regulations or something which was less formal and instead provided guidance on how to conduct ethical social media research. A *code of conduct* or *best practice* was supported by most participants to give reassurance to social media users and be helpful to researchers:

Some more national guidance on the ethics of social media research would be helpful. Because, it does seem unfortunate if everybody’s having to reinvent the wheel and different universities have different standards. You’d think that there would be some sort of national expectations on, you know maybe not hard and fast rules but at least guidance of best practice to actually make it easier for everybody. To make it easier for the researchers but also to make it easier for people who are using social media so that they know what people might get up.

Arabella, Female 31-46 Focus Group Participant

The logistics of regulation was also seen as a hindrance. It was thought that implementing any laws would be difficult, if not impossible to do. Questions were asked on how this could be policed or monitored and how, from an international perspective, this would work, as different countries would have different laws and social media data are available worldwide:

There should be [laws], but nothing's ever going to change...I can’t imagine a time when...anybody would be prosecuted for it, because who is going to keep looking at it?...If it can be policed properly or...I don’t think it can be. I don’t...who...you know, it’s national regulations, international, European, how do you get through it all, how do you get that? I don’t know. Yeah. It should be, but...I’m a...realist.

Joan, Female over 65 Interviewee

## Discussion

### Summary of Results

Our study provides information on members of the public’s views on the use of social media data for research, with a focus on adverse effects research. Opinion was divided, with some supportive of social media data being used freely as it was in the public domain, whereas others felt concerned about vulnerable groups, sensitive topics, and issues with people’s awareness of privacy regulations and how their data could be used. Our study found that participants were generally more positive about the study of adverse effects using social media data than the general use of social media in research. However, this support was caveated and dependent on a number of conditions being met. The most important condition was that social media data were used for research where there was clear societal benefit and users were protected from any potential harm that might arise from their data being used. The most powerful benefit to social media research for side effects was the potential to save lives.

The most alarming potential harm expressed was that by a virtual discussant who stated that they would not have used social media had they known researchers were trawling their posts. This is of great concern as social media have become a great source of support for many people, and this may be particularly the case for mental illness—where people are already very vulnerable [16].

There was divided opinion as to whether consent was required to use social media data for research purposes. This uncertainty could partly be attributed to the fact that many participants had neither realized that researchers used social media posts nor thought about any ethical issues, the *tricky* nature of social media research, and the different types of social media and research.

### Comparison With Existing Literature

We used a framework derived from a systematic review of the literature [8] to aid how we analyzed and interpreted the study’s findings. This framework evolved mostly from non–health-related research with an international coverage, which often included researcher’s views and those from social media users.

Many of the themes within this chosen framework are interrelated. For example, *self-regulation* is related to *harm*, as this leads to a decline in freedom and puts restraints on the supportive nature of social media. In addition, *self-regulation* is related to *privacy*, as people choose to keep certain information private, and *responsibilities*, as it puts the onus firmly in the hands of the social media user.

Most issues in this study were in line with previous study findings, regarding research using social media data in other areas, and this was demonstrated by the fact that no new themes emerged that were not already in the framework. But there were different issues raised within some of the themes—in particular, the responsibilities of social media moderators and governments were not emphasized as an issue in this study.

For instance, more emphasis was placed in this study on the beneficial aspects of social media research, validity of the social media data, and power of self-regulation than in previous studies. This is likely to be because of the potential societal benefit of adverse effects research, the perceived importance of high-quality research for adverse effects, and how increasingly savvy users are beginning to become in respect to lack of privacy on social media.

Unlike previous studies, in this study, no consideration was given to the potential harm to the researchers. This may be unsurprising given that the participants were more focused on the potential harms to social media users, as they were social media users themselves. The harms perceived for social media users in other types of research included bullying, abuse, and persecution, whereas here, the concerns in this study were more in line with preventing users from gaining support through heaving self-regulation or leaving sites. This is likely to be because of the international perspective of other studies and some of the topics (such as homosexuality and sexual abuse) covered. For instance, in some countries outside the United Kingdom, homosexuality is illegal or at least taboo.

### Implications

It is clear that social media users are in favor of some sort of overarching guidance for all institutions to follow. Our findings will not only help direct future research but will also provide social media websites, universities, ethics boards, pharma companies, and policymakers with evidence to inform policy and guidance on the use of social media data for research.

This research shows the variety of responses received when asking social media users about the ethics of using social media for a specific subject area. However, the complexity of ethical considerations is largely understood by social media users, as are the different considerations for different types of social media data collection, even within 1 research area. Although overall, there was support because of the large potential benefits, care must still be exercised when conducting social media research into adverse effects.

By harnessing technology, social media research can help inform research on adverse effects in a relatively easy and effective way. Although there may be no 1 rule that fits all regarding the ethical considerations, this research demonstrates the need for careful consideration of the ethics and increasing awareness of how social media are used in research. Participants expressed surprise at current practices in social media research or were unsure about what is currently being done with their posts or data and were eager to know more about privacy settings and how their data could be used. Education and information provision may be one of the most appropriate ways forward to help protect individuals. The use of patient and public involvement to develop consent and ethics processes for this research area may also help the approval processes for social media research. Thus, public and researchers could work together on an on-going basis, via means, such as committees or review panels.

The participants indicated that their opinions on the use of social media in research changed over time. Social media are constantly evolving communication means, with changing popularity between different platforms for different demographics. The use of people’s data is also increasingly becoming more apparent, particularly with recent news events. It is, therefore, imperative that the users of social media are consulted over time, as users reflect on new developments. Although this research demonstrates the value of ascertaining the views of users through interviews, virtual discussions, and focus groups, there is value in other types of research, such as Web-based surveys.

### Strengths and Limitations

The study adds to a limited qualitative evidence base on the use of social media data for research. The multi methods approach and range of recruitment strategies adopted ensured that a wide range of views were captured. Despite this, interview and focus group participants were predominately female and represented a limited geographical area. Our study also highlights the potential for using *virtual discussants*, a previously underutilized source of qualitative data collection, which can provide a viable source of qualitative data. However, when using this method in the future, researchers should be aware that obtaining permissions from social media platforms can limit access to these data, as was the case in this study, and because of the nature of the data collection and anonymity of these forums, gaining insight into the demographics of the participants can be difficult. Interviews and focus groups were also conducted by an academic researcher and a public health researcher. Although this may have influenced how participants responded to the idea of researchers using their social media data, criticisms and concerns were still provided.

### Conclusions

There appears to be a wide disparity in attitudes toward research using social media data from those who believe ethical approval (such as approval from an institutional review board, a research ethics board, or research governance body) is not necessary to those who support the idea that ethics should prevent such research taking place. Adverse effects are viewed as personal and, therefore, more likely to attract ethical consideration. However, adverse effects are also seen as an important area of research, which may have enormous benefit to society. All future adverse effects research should consider the ethical implications with an aim to minimize harm and maximize benefit. This research indicates the value that the public place on these aspects to aid researchers in the development of their research methods.

## Appendix

Multimedia Appendix 1 Interview topic guide.

Multimedia Appendix 2 Posts to create online discussion.

Multimedia Appendix 3 Focus group topic guide.

## Abbreviations

NHS: National Health Service
NIHR: National Institute for Health Research

## References

[1] Golder S, Norman G, Loke YK (2015). Systematic review on the prevalence, frequency and comparative value of adverse events data in social media. Br J Clin Pharmacol.

[2] Sarker A, Ginn R, Nikfarjam A, O'Connor K, Smith K, Jayaraman S, Upadhaya T, Gonzalez G (2015). Utilizing social media data for pharmacovigilance: a review. J Biomed Inform.

[3] Eysenbach G, Till JE (2001). Ethical issues in qualitative research on internet communities. Br Med J.

[4] Elgesem D (2002). What is special about the ethical issues in online research?. Ethics Inf Technol.

[5] Flicker S, Haans D, Skinner H (2004). Ethical dilemmas in research on internet communities. Qual Health Res.

[6] Heilferty CM (2011). Ethical considerations in the study of online illness narratives: a qualitative review. J Adv Nurs.

[7] Swirsky ES, Hoop JG, Labott S (2014). Using social media in research: new ethics for a new meme?. Am J Bioeth.

[8] Golder S, Ahmed S, Norman G, Booth A (2017). Attitudes toward the ethics of research using social media: a systematic review. J Med Internet Res.

[9] Capurro R, Pingel C (2002). Ethical issues of online communication research. Ethics Inf Technol.

[10] Whitehead LC (2007). Methodological and ethical issues in internet-mediated research in the field of health: an integrated review of the literature. Soc Sci Med.

[11] Herron M, Sinclair M, Kernohan WG, Stockdale DJ (2011). Ethical issues in undertaking internet research of user-generated content: a review of the literature. Evid Based Midwifery.

[12] Pope C, Mays N (2006). Qualitative Research in Health Care. Third Edition.

[13] Mikal J, Hurst S, Conway M (2016). Ethical issues in using Twitter for population-level depression monitoring: a qualitative study. BMC Med Ethics.

[14] Baker SE, Edwards R, Doidge M (2012). National Center for Research Methods.

[15] Braun V, Clarke V (2006). Using thematic analysis in psychology. Qual Res Psychol.

[16] Shepherd A, Sanders C, Doyle M, Shaw J (2015). Using social media for support and feedback by mental health service users: thematic analysis of a Twitter conversation. BMC Psychiatry.

