# Supplementary material for: Understanding Public Attitudes Toward Researchers Using Social Media for Detecting and Monitoring Adverse Events Data: Multi Methods Study
Source: J Med Internet Res. 2019 Aug 29;21(8):e7081. doi: 10.2196/jmir.7081 (PMC6740159; doi:10.2196/jmir.7081)
Supplement: Multimedia Appendix 1 [file jmir_v21i8e7081_app1.pdf]

Interview Topic Guide

Attitudes towards researchers using social media for detecting and monitoring side effects of treatments

Introduction

Thank you very much for taking part in this study. My name is Su Golder and I work at the University of York. As you know, we are carrying out some research on how people feel about researchers using social media for detecting and monitoring side effects of treatments. Lots of people use social media (such as Twitter, Facebook and discussion forums) to post information on side effects that they or someone they know have experienced.

Potentially researchers interested in the side effects of a particular drug or treatment can search social media using sophisticated technology to find these posts.

This interview is very informal and completely confidential. Only my colleagues and I will see it and your name will not appear in anything we write.

With your permission I would like to record the interview. This is so that I can concentrate on what you are telling me rather than spending the whole time taking notes. Is that OK? The recordings will be stored securely and destroyed after 3 years after the study is complete.

Before we begin do you have any questions?

Main Questions

Background about social media use

To start off, I would like to find out a little bit about your experiences of social media

So can I start by asking you which social media sites you use? How often do you use social media sites? How often d[illegible] post on social media? What do you use social media for?

Health information online

Do you ever look up health information on social media? Why or why not?

Do you ever post or discuss health issues online?

Reporting of side effects (side effects are effects that are in addition to the intended effect of a treatment and c[illegible] harmful or unpleasant)

Have you ever posted or mentioned a side effect of a drug or other treatment on social media?

Have you ever told a health professional, such as a doctor or nurse about a side effect of a drug or other treatment?

Have you ever told a regulatory agency (for instance by using the Yellow Card scheme in the UK), or a drug compa[illegible] manufacturer about a side effect of a drug or other treatment?

Have you ever told anyone else about a side effect of a drug or other treatment?

Attitudes towards researchers using social media (Some researchers are using social media to carry out their rese[illegible] for example, looking up opinions or forecasting general elections. This is now becoming increasingly comm[illegible] healthcare research so researchers can predict disease outbreaks or find out about patterns of drug use)

How do you feel about researchers using social media posts to look at health information, such as monitoring t[illegible] epidemic or illegal drug use?

How do you feel about researchers using social media posts to identify side effects? Is it different to other typ[illegible] information?

Different types of research or researchers

Does it make a different who is doing the monitoring for side effects?

Do you feel differently about say an academic from a university, a charity, the NHS, a private company (such as the[illegible] company or its competitors) or government body?

Does it make a difference which social media are used for monitoring side effects?

Do you feel differently about different social media platforms (public vs private, Twitter vs Facebook, Health discu[illegible] forums, chatrooms)?

Privacy expectations

Do you know who is able to view or monitor your social media posts?

Some researchers have said that using social media data poses no ethical issues because users understand there privacy in social media. How do you feel about that?

Do you expect researchers to ask for your permission to use your posts?

What about if the researcher wants to count the number of posts with a specific side effect? E.g. '58 people postec they suffered from weight gain after receiving drug A'

Or what about if the researcher wants to quote you describing a side effect? For example, 'I've started taking drug A it has made be put on loads of weight'.

If permission is required, how do you think the researcher should gain this? Should they try to contact you and ask Should individual sites ask if you are happy for your posts to be part of research? Could something be included in the t of service that you agree to when you use the site?

Should they disclose they are a researcher? For example, in chatrooms or discussion forums.

Do you expect to be made anonymous if your post is used in research? Is it enough for researchers to exclude your na Or should they remove all identifying clues from any posts quoted in their reports or publications?

If you are quoted do you want to be named and given credit for your post?

Research Conduct

If you agree that social media can be used for identifying and monitoring side effects, how should such data be colle and used? For example, do you think there should be any conditions or terms that need to be followed? Are there laws or rules you would like to see followed?

Anything Else

Is there anything else you would like to add?

Can I ask which age category you belong to 18-30, 31-45,46-65, 65+

Thank you very much for taking part.

If you would like a summary copy of this research please let me know.
