# Supplementary material for: Understanding Public Attitudes Toward Researchers Using Social Media for Detecting and Monitoring Adverse Events Data: Multi Methods Study
Source: J Med Internet Res. 2019 Aug 29;21(8):e7081. doi: 10.2196/jmir.7081 (PMC6740159; doi:10.2196/jmir.7081)
Supplement: Multimedia Appendix 2 [file jmir_v21i8e7081_app2.pdf]

Social Media Discussions

Email to moderator:

My name is Dr Su Golder and I work at the University of York. We are carrying out some research on how people feel about researchers using social media for detecting and monitoring side effects of treatments. Lots of people use social media (such as Twitter, Facebook and discussion forums) to post information on side effects that they or someone they know have experienced.

Potentially researchers interested in the side effects of a particular drug or treatment can search social media using sophisticated technology to find these posts.

We would like to start a thread using the following post "If this social media site were to be used by researchers to identify information on the side effects of medications how would you feel?"

I would first like to ask your permission.

Post to create online discussions

In instances where permission was granted the following text was posted on the social media site and users were free to post and join the resulting discussion. "Attitudes towards researchers using social media for detecting and monitoring side effects of treatments

My name is Dr Su Golder and I work at the University of York. We are carrying out some research on how people feel about researchers using social media for detecting and monitoring side effects of treatments. Lots of people use social media (such as Twitter, Facebook and discussion forums) to post information on side effects that they or someone they know have experienced.

Potentially researchers interested in the side effects of a particular drug or treatment can search social media using sophisticated technology to find these posts.

We would like to hear the views of social media users. For more information on our research please email me at su.golder@york.ac.uk.

If you would like to participate in this research please respond on this social media site to the question below. Any responses may be used in our research (for a copy of our participant information sheet – please email me at su.golder@york.ac.uk).

If this social media site were to be used by researchers to identify information on the side effects of medications how would you feel?"
