# Supplementary material for: Understanding Public Attitudes Toward Researchers Using Social Media for Detecting and Monitoring Adverse Events Data: Multi Methods Study
Source: J Med Internet Res. 2019 Aug 29;21(8):e7081. doi: 10.2196/jmir.7081 (PMC6740159; doi:10.2196/jmir.7081)
Supplement: Multimedia Appendix 3 [file jmir_v21i8e7081_app3.pdf]

Focus Group Topic Guide

Attitudes towards researchers using social media for detecting and monitoring side effects of treatments

Introduction

Thank you very much for taking part in this study. My name is Su Golder and I work at the University of York. As you know, we are carrying out some research on how people feel about researchers using social media for detecting and monitoring side effects of treatments. Lots of people use social media (such as Twitter, Facebook and discussion forums) to post information on side effects that they or someone they know have experienced.

Potentially researchers interested in the side effects of a particular drug or treatment can search social media using sophisticated technology to find these posts.

This focus group is very informal and completely confidential. Only my colleagues and I will see it and your name will not appear in anything we write.

With your permission I would like to record the focus group. This is so that I can concentrate on what you are discussing rather than spending the whole time taking notes. Is that OK? The recordings will be stored securely and destroyed after 3 years after the study is complete.

Before we begin do you have any questions?

Scenarios

Six scenarios are presented below. Please think about how you feel about each scenario and whether you agree to social media posts being used in each scenario.

If there are any conditions or caveats which you would like to see met before the social media can be used in this please discuss these.

Scenario A<br>An academic researcher wants to look up the numbers of side effects of a drug they are studying. They intend to search on Twitter.
Scenario B<br>A private drug company wants to look up the numbers of side effects of a drug they produce and a competitor drug made by a different drug company. They intend to use patient forums.
Scenario C<br>A charity wants to use quotes posted on social media to highlight side effects of a drug used for the disease the charity provides support for. They propose to use all social media sites (from closed patient forums to Twitter).
Scenario D<br>A government agency wants to use quotes posted on social media to highlight side effects of a drug used to support a political decision. They propose to use all social media sites (from closed patient forums to Twitter).
Scenario E<br>An academic researcher wants to use quotes posted on social media to highlight side effects of a drug to support the results of a research study. They propose to use all social media sites (from closed patient forums to Twitter).
Scenario F<br>A private drug company wants to use quotes posted on social media to highlight side effects of a competitor drug in an advertisement product. They intend to use Twitter.

Statement 1<br>You should never look up health information on social media.
Statement 2<br>I am happy to discuss personal issues or health issues on the internet.
Statement 3<br>Research using social media data poses no ethical issues because the data are already public.
Statement 4<br>I know who is able to view or monitor my social media posts.
Statement 5<br>Researchers need to ask permission to use people's posts in their research.
Statement 6<br>If I was quoted in a research study I would want to be made anonymous.
Statement 7<br>Researchers do not need to disclose that they are a researcher.
Statement 8<br>There should be laws for researchers to follow when using social media posts in their research.
